# Refractive and Vector Outcomes of SMILE Pro with the VISUMAX 800 for High Astigmatism

**DOI:** 10.3390/vision10030045

**Published:** 2026-07-17

**Authors:** Lan Huong Thi Tran, Thanh Ngoc Tran, Hong Son Cung, Khanh-Sam Tran, Van Trong Pham

**Affiliations:** 1 Department of Ophthalmology and Optometry, Hanoi Medical University, Hanoi 11520, Vietnam; 2 Vietnam National Eye Hospital, Hanoi 11626, Vietnam; dr.thanhntran@gmail.com (T.N.T.);; 3 Department of General Ophthalmology, Cung Hong Son International Eye Hospital, Hanoi 11715, Vietnam; cunghongson@benhvienmathongson.com

**Keywords:** astigmatism, keratorefractive lenticule extraction, refractive surgery, SMILE Pro, vector analysis, VISUMAX 800

## Abstract

**Background/Objectives:** High astigmatism remains challenging in keratorefractive lenticule extraction because small errors in centration or cyclotorsion may affect astigmatic correction. This study evaluated early refractive and astigmatic vector outcomes after small-incision lenticule extraction (SMILE) Pro using the VISUMAX 800 in eyes with high astigmatism. **Methods:** This prospective interventional cohort study was conducted at Cung Hong Son International Eye Hospital, Hanoi, Vietnam. Refractive outcomes, including uncorrected distance visual acuity (UDVA), corrected distance visual acuity (CDVA), manifest refraction, astigmatic vector analysis, and intraoperative and postoperative complications, were evaluated up to 3 months postoperatively. **Results:** A total of 160 eyes from 102 patients were included in the final analysis. The mean age was 23.7 ± 4.7 years, and 63.7% of participants were female. The mean preoperative spherical equivalent (SEQ) was −7.21 ± 2.03 D, and the mean preoperative refractive cylinder was −2.51 ± 0.56 D. Mean postoperative SEQ was −0.10 ± 0.38 D at 1 month and 0.02 ± 0.31 D at 3 months. At 3 months, 99% and 84% of eyes achieved UDVA of 0.8 decimal or better and 0.9 decimal or better, respectively. The mean residual refractive cylinder was −0.52 ± 0.28 D, with 69% and 98% of eyes achieving a residual cylinder ≤0.50 D and ≤1.00 D, respectively. All eyes had an angle of error within ±15°. The safety index was 1.01, and no eyes lost two or more lines of CDVA. **Conclusions:** SMILE Pro performed with the VISUMAX 800 provided favorable early 3-month refractive and astigmatic vector outcomes with a satisfactory safety profile in eyes with high astigmatism.

## 1. Introduction

The volume of refractive surgery has increased steadily over the past decade, with an estimated annual growth rate of approximately 9.6% between 2020 and 2025 [1]. Recent Google Trends data from Taiwan, Hong Kong, Singapore, and global searches indicate growing interest in SMILE and implantable collamer lens, while interest in LASIK has declined in several regions [2]. Keratorefractive lenticule extraction (KLEx) was introduced in 2011 and has been widely adopted because of its favorable biomechanical profile, reduced incidence of postoperative dry eye symptoms, rapid visual recovery, and high refractive accuracy [3,4,5,6]. Nevertheless, the correction of high astigmatism (≥2.00 D) with KLEx remains technically challenging. Limitations related to intraoperative cyclotorsion, head positioning, and the absence of active eye-tracking or automated centration systems have been associated with a tendency toward undercorrection in higher cylindrical treatments [7,8,9,10].

The latest generation of this technology, SMILE Pro performed using the VISUMAX 800 (Carl Zeiss Meditec, Jena, Germany), incorporates a higher laser repetition rate of up to 2 MHz, as well as upgraded software designed to enhance centration and minimize cyclotorsional misalignment. Although early clinical studies have demonstrated favorable outcomes in the treatment of myopic astigmatism, evidence specifically addressing refractive accuracy and astigmatic vector outcomes in eyes with high astigmatism remains limited. Therefore, this study aimed to evaluate the refractive and vector outcomes of SMILE Pro in the correction of high astigmatism.

## 2. Materials and Methods

### 2.1. Study Design and Patients

This prospective interventional cohort study was conducted at Cung Hong Son International Eye Hospital (Hanoi, Vietnam) and approved by the Institutional Review Board of Hanoi Medical University (IRB ID: HMUIRB1649). The study was retrospectively registered at ClinicalTrials.gov after participant enrollment had begun but before data analysis (NCT07446751; registration date: 10 February 2026; Unique Protocol ID: HMUIRB1649). Retrospective registration was performed because public trial registration was completed after the start of enrollment. However, the study protocol had been approved by the Institutional Review Board before study conduct, and trial registration was completed before data analysis and manuscript preparation. Although the registered study includes planned follow-up to 12 months, the present manuscript reports early 3-month refractive and vector outcomes from this ongoing prospective study. The study adhered to the tenets of the Declaration of Helsinki. All participants received detailed explanations regarding the surgical procedure and the study protocol and provided written informed consent for both treatment and the use of their clinical data for research purposes.

Patients aged 18 to 40 years who underwent SMILE Pro for the correction of high astigmatism between 1 December 2024 and 31 December 2025 were enrolled and followed for up to 3 months postoperatively. All preoperative and postoperative examinations were performed by an experienced optometrist using standardized measurement protocols. Surgical procedures were performed by a single refractive surgeon to minimize inter-surgeon variability. Inclusion criteria were myopia up to −10.00 diopters (D) with astigmatism from 2.00 to 5.00 D, stable refraction defined as a change of no more than 0.50 D within the preceding 6 months, and discontinuation of soft contact lenses for at least 1 week and rigid gas-permeable lenses for at least 4 weeks prior to evaluation. Preoperative corrected distance visual acuity (CDVA) was required to be 20/25 (0.10 logMAR/0.8 decimal) or better. The estimated residual stromal thickness (RST) had to be ≥250 µm. Exclusion criteria were previous ocular surgery, irregular corneal tomography, keratoconus or keratoconus suspect, cataracts, glaucoma, active vitreoretinal disease, active keratitis, or severe dry eye disease (tear breakup time <5 s according to JDES/ADES criteria) [11]. Additional exclusions were monocular status, systemic diseases that could affect wound healing or refractive stability such as diabetes mellitus or autoimmune disorders, pregnancy, lactation, and the use of hormonal therapies.

### 2.2. Preoperative and Postoperative Examinations

All patients underwent a comprehensive ophthalmic examination, including uncorrected distance visual acuity (UDVA), manifest and cycloplegic refraction, CDVA, slit-lamp biomicroscopy, dilated fundus examination, and intraocular pressure measurement. Visual acuity measurements were recorded in decimal. Biometric measurements were obtained using the IOLMaster 700 (Carl Zeiss Meditec, Jena, Germany), and corneal tomography was performed with the Sirius CSO+ (CSO, Florence, Italy). Postoperative examinations were scheduled at 1 week, 1 month, and 3 months. At each visit, UDVA, CDVA, manifest refraction, slit-lamp examination findings, and corneal tomography were recorded. Patients were prescribed moxifloxacin 0.5%, fluorometholone 0.1% and sodium hyaluronate 0.3% eyedrops postoperatively.

### 2.3. Surgical Technique

All procedures were performed using the VISUMAX 800 femtosecond laser system (Carl Zeiss Meditec, Jena, Germany). The cap thickness was set at 100 µm, with a cap diameter ranging from 7.50 to 7.80 mm. The optical zone ranged from 6.00 to 6.80 mm, depending on individual refractive parameters. Laser settings included a pulse energy of 140 nJ, spot spacing of 4.4 µm, and track spacing of 3.8 µm for both cap and lenticule creation. Surgical planning was primarily based on stable preoperative manifest refraction, with cycloplegic refraction used as a confirmatory measurement. If manifest and cycloplegic findings were not clinically concordant, surgery was deferred and refraction was reassessed before final treatment planning. The intended postoperative target was plano in all eyes.

Preoperative biometric data from the IOLMaster 700 were transferred to the VISUMAX 800 to enable iris registration. The integrated OcuLign Auto^®^ and CentraLign^®^ systems were used intraoperatively to optimize centration and reduce cyclotorsion misalignment under real-time surgeon guidance. No manual preoperative corneal marking was performed. A surgeon-adjusted cylindrical nomogram, incorporating an additional 10% of the intended cylindrical correction, was defined before the start of this high-astigmatism cohort and applied uniformly to all eyes throughout the study period. This adjustment was based on prior institutional outcomes with SMILE Pro using the VISUMAX 800, in which astigmatic undercorrection tended to increase with higher preoperative cylinder magnitude, with clearer undercorrection observed in eyes with a preoperative cylinder greater than 2.50 D [12]. The same 10% adjustment was applied regardless of cylinder magnitude, astigmatic axis, optical zone diameter, or spherical equivalent, and the nomogram was not modified during enrollment. Lenticule centration and intraoperative cyclotorsion parameters were recorded automatically by the VISUMAX 800 for subsequent analysis.

A small 2-mm incision was created at the 120° position, with an incision angle of 29° and a side-cut angle of 90°. The lenticule was dissected using a Chansue dissector and extracted through the small corneal incision according to standard SMILE technique.

### 2.4. Statistical Analysis

The sample size was calculated using the formula for a single proportion, based on a previous report in which 92% of eyes achieved UDVA of 20/20 or better after SMILE in patients with high astigmatism (≥2.00 D) [8]. Assuming a significance level of 0.05, an expected proportion of 0.92, and a precision of 0.05, the minimum required sample size was 113 eyes. Because limited prior data were available for astigmatic vector endpoints after SMILE Pro in eyes with high astigmatism, the sample size calculation was based on this clinically relevant visual efficacy endpoint. Astigmatic vector outcomes were prespecified as key secondary outcomes, and subgroup analyses were interpreted cautiously because of the imbalanced distribution of preoperative astigmatism severity. Lenticule decentration was assessed as the radial offset between the planned lenticule center and the corneal vertex, as automatically recorded by the VISUMAX 800. Mathematically, radial decentration may be expressed as d = $\sqrt{x^{2}+y^{2}}$, where x and y correspond to horizontal and vertical displacement components. In the exported surgical dataset used for the present analysis, decentration was available only as rounded radial magnitude in 0.1-mm increments, and separate *x*- and *y*-axis components were not included. Therefore, decentration was analyzed as radial magnitude rather than directional displacement. For descriptive reporting, recorded values were grouped as 0.0 mm, 0.1 mm, and ≥0.2 mm, reflecting the exported data format rather than exact continuous intervals or predefined biological cutoffs. For regression analyses, lenticule decentration magnitude was analyzed as a continuous variable scaled per 0.1 mm.

Intraoperative cyclotorsion was recorded in degrees, with negative values indicating clockwise rotation and positive values indicating counterclockwise rotation. For correlation and regression analyses, the absolute value of cyclotorsion was used to represent the magnitude of rotational deviation, while directional distribution was summarized descriptively. Visual acuity was rounded to the closest line (more than half the letters on the line) for analysis and graphic representation.

The primary analysis focused on 3-month refractive outcomes. Data from earlier follow-up visits (1 week and 1 month) were summarized descriptively. Statistical analyses were performed using SPSS software (version 20.0; IBM Corp., Armonk, NY, USA). Because some patients contributed both eyes to the study, generalized estimating equations (GEE) were applied to account for inter-eye correlation, assuming an exchangeable working correlation structure. Robust standard errors were used in all models. Multivariable GEE models were constructed to identify factors associated with 3-month difference vector, absolute angle of error, and residual refractive cylinder magnitude. Predictors included age, sex, preoperative spherical equivalent, preoperative cylinder magnitude, optical zone diameter, estimated residual stromal thickness, absolute intraoperative cyclotorsion, and lenticule decentration magnitude. Optical zone diameter and lenticule decentration were scaled per 0.1 mm, and estimated residual stromal thickness was scaled per 10 µm. All predictors were entered simultaneously, without automated stepwise selection. Additional model estimates are provided in Supplementary Table S1. Visual and refractive outcomes were presented according to the Standard Graphs for Reporting Refractive Surgery [13] and astigmatic vector analysis was performed using the Alpins method with ASSORT software (version 1.1.4; ASSORT Pty. Ltd., Cheltenham, VIC, Australia) [14].

For Alpins vector analysis, target-induced astigmatism (TIA) was defined using the preoperative manifest refractive cylinder, rather than the nomogram-adjusted laser-programmed cylinder. This approach was chosen because the intended clinical refractive target was plano, and the purpose of treatment was to neutralize the preoperative refractive astigmatism. The additional 10% cylindrical nomogram was applied during laser treatment planning as a compensation strategy for expected undercorrection, but it was not used to redefine the clinical target-induced astigmatism in the vector analysis.

With respect to missing intermediate follow-up data, available-case analysis was applied. Eyes with complete baseline and 3-month data were included in the primary analyses. No imputation was performed for missing values.

## 3. Results

A total of 160 eyes from 102 patients were included in the final analysis. The mean age was 23.7 ± 4.7 (18 to 40 years) and 63.7% of participants were female. Baseline demographic and clinical characteristics are summarized in Table 1. All eyes completed the 3-month follow-up. Interim follow-up data were available for 143 eyes at the 1-week visit and 103 eyes at the 1-month visit. Preoperative astigmatism was categorized into predefined magnitude subgroups (Table 2), and all eyes presented with with-the-rule astigmatism.

### 3.1. Accuracy and Predictability

**Figure 1:**
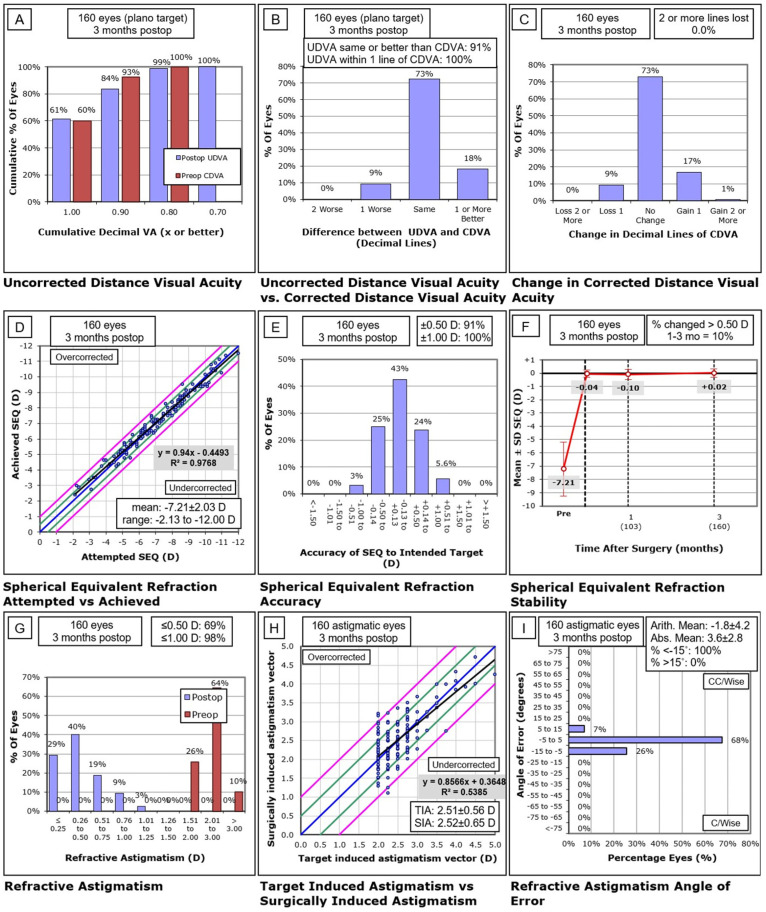
Standard graphs for refractive outcomes after SMILE Pro with the VISUMAX 800 in eyes with high astigmatism. (**A**) Cumulative uncorrected distance visual acuity at 3 months compared with preoperative corrected distance visual acuity. (**B**) Difference between postoperative uncorrected distance visual acuity and preoperative corrected distance visual acuity. (**C**) Change in corrected distance visual acuity. (**D**) Attempted versus achieved spherical equivalent refraction. (**E**) Accuracy of spherical equivalent refraction relative to intended target. (**F**) Stability of spherical equivalent refraction. (**G**) Distribution of preoperative and postoperative refractive astigmatism. (**H**) Target-induced astigmatism versus surgically induced astigmatism. (**I**) Distribution of refractive astigmatism angle of error.

Refractive outcomes at 3 months postoperatively showed that UDVA was 0.8 decimal or better in 99% and 0.9 decimal or better in 84% of eyes (Figure 1A). Postoperative UDVA was equal to or better than preoperative CDVA in 91% of eyes (Figure 1B). The efficacy index was 0.99, calculated from a mean postoperative UDVA of 0.03 ± 0.04 logMAR and a mean preoperative CDVA of 0.02 ± 0.03 logMAR. Regarding refractive accuracy, attempted versus achieved SEQ showed a strong linear relationship (Figure 1D), and the achieved SEQ was within ±0.25 D in 71% of eyes, within ±0.50 D in 91%, and within ±1.00 D of target in 100% of eyes (Figure 1E).

### 3.2. Stability

The mean preoperative SEQ was −7.21 ± 2.03 D (range, −2.13 to −12.00 D). At the 1-week postoperative visit, the mean SEQ was −0.04 ± 0.29 D (*n* = 143). At 1 month, the mean SEQ was −0.10 ± 0.38 D (*n* = 103), and at 3 months, it was 0.02 ± 0.31 D (*n* = 160) (Figure 1F).

The mean residual refractive cylinder was −0.51 ± 0.29 D at the 1-month visit and −0.52 ± 0.28 D at the 3-month visit. When stratified by preoperative astigmatism magnitude, no significant difference in the residual cylinder was observed among preoperative astigmatism subgroups (Wald χ^2^ = 0.665, df = 2, *p* = 0.717). Table 3 shows additional analysis of astigmatism stability by magnitude subgroup. There was no clinically significant change in refractive astigmatism between the 1-month and 3-month visits regardless of magnitude. At 3 months, a residual refractive cylinder ≤0.50 D was achieved in 95/136 eyes (69.9%) in Group 1, 11/17 eyes (64.7%) in Group 2, and 5/7 eyes (71.4%) in Group 3.

### 3.3. Safety

At 3 months, 73% of eyes showed no change in CDVA compared with preoperative values. A gain of one line was observed in 17% of eyes, and a gain of two or more lines was observed in 1%. Nine percent of eyes lost one line of CDVA, and no eyes lost two or more lines (Figure 1C). The safety index was 1.01. Minor epithelial tears at the main incision occurred in 6.25% of cases, with no associated delay in healing. No other intraoperative or postoperative complications were observed.

### 3.4. Astigmatism Analysis

**Figure 2:**
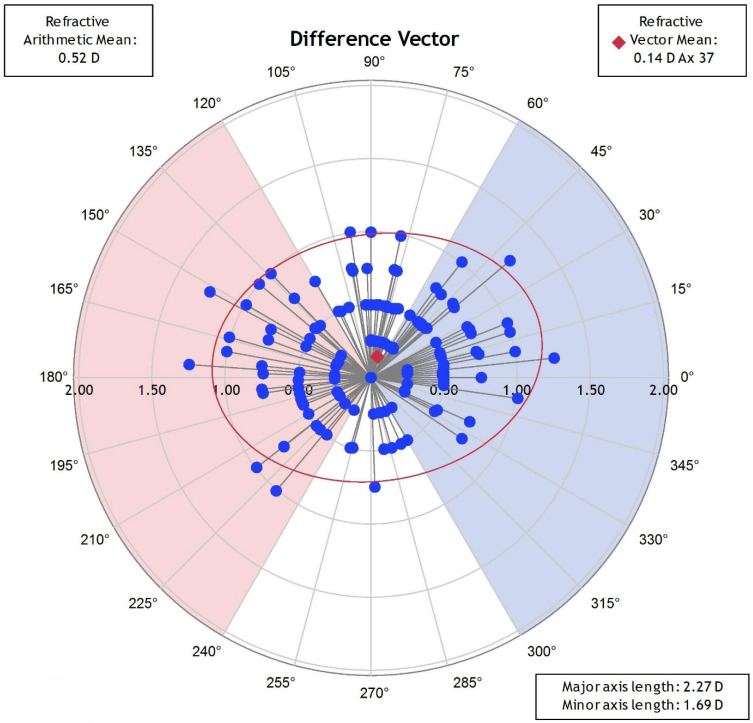
Polar plot of the difference vector at 3 months after SMILE Pro with the VISUMAX 800 in eyes with high astigmatism. Each blue point represents the difference vector of an individual eye, with radial distance indicating vector magnitude in diopters (D) and angular position indicating axis orientation in degrees. The red diamond denotes the refractive vector mean. A vector mean close to the origin indicates limited net systematic residual astigmatic bias. The ellipse illustrates the dispersion of individual difference vectors around the vector mean, with the major and minor axis lengths shown in diopters.

At 3 months, 69% of eyes had a residual refractive cylinder ≤0.50 D and 98% had a residual refractive cylinder ≤1.00 D (Figure 1G). The mean absolute intraoperative cyclotorsion recorded by the VISUMAX 800 was 3.62 ± 2.19° (range, 0 to 10°). Directional cyclotorsion was clockwise in 116 eyes (72.5%), counterclockwise in 43 eyes (26.9%), and absent in 1 eye (0.6%). The mean lenticule decentration magnitude was 0.14 ± 0.09 mm (range, 0.00 to 0.50 mm). Based on the rounded radial decentration values available in the exported surgical dataset, recorded decentration was 0.0 mm in 28 eyes (17.5%), 0.1 mm in 121 eyes (75.6%), and ≥0.2 mm in 11 eyes (6.9%). Vector analysis using the Alpins method showed a mean absolute angle of error of 3.6 ± 2.8°, with 100% of eyes having an angle of error within ±15° (Figure 1I). Scatter plots of surgically induced astigmatism versus target-induced astigmatism demonstrated a positive linear relationship (Figure 1H). The mean correction index (CI) was 1.01 ± 0.23, indicating overall accurate cylindrical correction. The mean difference vector (DV) was 0.52 ± 0.28 D (Figure 2).

In multivariable GEE models, greater preoperative cylinder magnitude was independently associated with greater absolute angle of error at 3 months (B = 0.95 per 1 D increase; *p* = 0.023), but not with difference vector or residual refractive cylinder magnitude (Table 4). No significant associations were observed between the remaining evaluated predictors and the main astigmatic vector or residual refractive outcomes. Additional model estimates are provided in Supplementary Table S1.

## 4. Discussion

This prospective interventional cohort study evaluated early refractive and astigmatic vector outcomes after lenticule extraction with the VISUMAX 800 in eyes with high preoperative astigmatism (≥2.00 D). SMILE Pro showed favorable efficacy and refractive accuracy, with 91% and 100% of eyes achieving SEQ within ±0.50 D and ±1.00 D of target, respectively. The residual refractive cylinder was also favorable in this high-astigmatism cohort, with 69% of eyes achieving ≤0.50 D and 98% achieving ≤1.00 D at 3 months. Refractive astigmatism remained stable between 1 and 3 months after surgery.

The results of the current study are consistent with previous reports of SMILE Pro for compound myopic astigmatism. Reinstein et al. demonstrated postoperative astigmatism ≤0.50 D in 84.2% of eyes, a correction index of 1.05, and an absolute angle of error within ±15° in 81% of eyes [15]. However, that study included a relatively small cohort with a lower mean preoperative refractive cylinder (−0.98 ± 0.78 D). In a multicenter clinical study of myopic astigmatism, Sekundo et al. reported a postoperative refractive cylinder ≤0.50 D in 90.8% of eyes, with 86% of eyes showing an angle of error within ±15° [16]. Although limbal marking and contact glass rotation under OcuLign guidance were used, a slight undercorrection was observed when surgically induced astigmatism was compared with target-induced astigmatism. The authors also suggested that higher preoperative cylinder magnitude may be associated with a broader distribution of angle of error. In our previous study of SMILE Pro for myopic astigmatism, the postoperative residual cylinder was ≤0.50 D in 78% of eyes, and 83% of eyes had an absolute angle of error within ±15°. A tendency toward undercorrection was observed in eyes with a preoperative cylinder greater than 2.50 D, which was attributed to the absence of nomogram adjustment at that time [12]. The present study included eyes with a higher preoperative SEQ and cylinder than those in previous reports and used a modified nomogram with additional cylindrical correction. Despite the greater refractive severity, the outcomes remained favorable, supporting the short-term clinical performance of this treatment approach in eyes with high astigmatism.

Recently, lenticule extraction using alternative femtosecond laser systems has been introduced, including Smart-Sight (Schwind ATOS, Schwind) and SILK (ELITA, Johnson & Johnson). Pradhan et al. reported that 60% of eyes achieved a spherical equivalent within ±0.50 D and 87% had residual astigmatism ≤0.50 D, with a correction index of 1.07 ± 0.28 [17]. Similarly, Sachdev et al. evaluated 170 eyes that underwent the SILK procedure and found that the postoperative mean SEQ was within ±0.50 D in 93.5% of eyes at 3 months, and that 96% of eyes achieved UDVA of 20/20 or better [18]. However, these studies included eyes with a lower preoperative cylinder and relatively small sample sizes. Further studies are needed to clarify the efficacy of Smart-Sight and SILK for high astigmatism correction.

To the best of our knowledge, this is among the first prospective studies specifically evaluating SMILE Pro for preoperative astigmatism of 2.00 D or greater. Lee et al. and Varman et al. compared outcomes of lenticule extraction using the VisuMax and VISUMAX 800 in eyes with compound myopic astigmatism and a cylinder greater than 1.50 D [19,20]. Both studies reported improved astigmatic accuracy with the VISUMAX 800, including a lower postoperative residual cylinder at 3 months (0.50 ± 0.41 D and 0.14 ± 0.29 D, respectively), lower mean difference vector (0.76 and 0.15, respectively), and higher correction index (0.83 and 0.98, respectively). In the current study, astigmatic correction was similarly accurate, with a mean correction index of 1.01 ± 0.23 and a mean difference vector of 0.52 ± 0.28 D.

Subgroup analysis showed comparable residual cylinder control across preoperative astigmatism subgroups, with 69.9%, 64.7%, and 71.4% of eyes in Groups 1, 2, and 3, respectively, achieving a residual refractive cylinder ≤0.50 D at 3 months. In addition, all three subgroups showed stable mean refractive astigmatism between 1 and 3 months postoperatively (Table 3).

In the multivariable GEE analysis, greater preoperative cylinder magnitude was associated with greater absolute angle of error (B = 0.95 per 1 D increase; *p* = 0.023), whereas no significant association was observed with difference vector or residual refractive cylinder magnitude. This finding suggests that eyes with higher baseline astigmatism may be more sensitive to small axis-related deviations, even when the overall residual astigmatic magnitude remains low. The absence of significant associations between optical zone diameter, absolute cyclotorsion, lenticule decentration, and the main astigmatic outcomes should be interpreted cautiously in the context of the single-arm design, use of intraoperative alignment assistance, and predefined cylindrical nomogram. Together, these findings underscore the need to consider baseline astigmatic magnitude, intraoperative alignment parameters, and the applied nomogram together when interpreting outcomes in high-astigmatism SMILE Pro, while avoiding attribution of the favorable results to any single platform-related factor.

This study has several limitations. First, the follow-up period was relatively short. The registered study includes planned follow-up to 12 months, whereas the present manuscript reports early 3-month refractive and vector outcomes. Continued follow-up of this cohort is ongoing and will allow assessment of longer-term refractive stability, visual quality, and late complications. Although previous studies have demonstrated that lenticule extraction outcomes are generally stable between 3 and 12 months, longer follow-up remains warranted in eyes with high astigmatism [5,21]. Second, this was a prospective single-center, single-surgeon, single-arm cohort without a comparator group. In addition, some participants contributed both eyes to the analysis. Although inter-eye correlation was addressed using generalized estimating equations with robust standard errors, inclusion of bilateral eyes may limit direct comparability with studies that include only one eye per participant. Thus, the findings should be interpreted as early clinical outcomes rather than evidence of superiority over previous SMILE platforms, other lenticule extraction systems, or alternative astigmatic correction strategies. Moreover, all eyes were treated using a predefined surgeon-adjusted cylindrical nomogram that incorporated an additional 10% of the intended cylindrical correction. Accordingly, the favorable astigmatic outcomes observed in this cohort should be interpreted in the context of this nomogram and should not be attributed solely to the VISUMAX 800 platform or its alignment systems. Because Alpins vector analysis used the manifest refractive cylinder as the clinical target-induced astigmatism, vector outcomes should be regarded as clinical refractive vector outcomes obtained under this nomogram-based treatment strategy, rather than as isolated measures of the laser-programmed cylindrical input. Third, the astigmatism subgroups were imbalanced, with most eyes in the 2.00 to 2.75 D subgroup and relatively few eyes with a cylinder of 3.00 D or greater. Therefore, subgroup findings in eyes with higher degrees of astigmatism should be interpreted cautiously. Fourth, all eyes had with-the-rule astigmatism, which may limit the generalizability of the findings to eyes with oblique or against-the-rule astigmatism. Fifth, visual acuity was measured using a decimal chart, which may lead to slight overestimation of true visual acuity when converted to logMAR or Snellen notation [22,23]. This limitation is likely to be more pronounced in eyes with poorer visual acuity. Decimal steps also do not correspond exactly to equal logarithmic intervals, which may introduce minor rounding errors during analysis. For this reason, visual acuity results were reported in decimal where possible. Finally, because the main objective of this study was to evaluate refractive predictability, safety, and astigmatic vector correction, functional visual quality endpoints such as contrast sensitivity and patient-reported quality of vision were outside the scope of the current analysis. All measurements were obtained using a standardized examination protocol. Future studies with longer follow-up, comparator groups, larger numbers of eyes with very high astigmatism, broader functional assessments, and evaluation of corneal biomechanical behavior may help confirm long-term stability, refine laser parameters, and improve outcomes in eyes with high astigmatism.

In conclusion, SMILE Pro with the VISUMAX 800 provided favorable early 3-month refractive and astigmatic vector outcomes in eyes with high preoperative astigmatism, most of which were in the 2.00 to 2.75 D range. These findings suggest good short-term predictability and safety when using a predefined surgeon-adjusted cylindrical nomogram. Longer follow-up, larger numbers of eyes with very high astigmatism, and comparative studies are needed to confirm refractive stability and generalizability.

## Figures and Tables

**Table 1:** Baseline Demographic and Clinical Characteristics.

Characteristics	Values
Age (years)	23.7 ± 4.7 (18 to 40)
Female, *n* (%)	65 (63.7)
Sphere (D)	−6.00 ± 2.00 (−0.50 to −10.00)
Cylinder (D)	−2.51 ± 0.56 (−2.00 to −5.00)
Spherical equivalent (D)	−7.21 ± 2.03 (−2.13 to −12.00)
UDVA (logMAR)	1.4 ± 0.16 (0.4 to 1.7)
CDVA (logMAR)	0.02 ± 0.03 (0.0 to 0.1)
CCT (µm)	549.9 ± 30 (481 to 629)
Estimated RST (µm)	299.9 ± 26.9 (268 to 378)
OZ (mm)	6.63 ± 0.27 (6.0 to 6.8)
IOP (mmHg)	16.1 ± 2.75 (10 to 20)
Mesopic pupil size (mm)	6.23 ± 0.78 (3.43 to 8.23)

Values are presented as the mean ± SD (range) unless otherwise indicated. Age and sex are patient-level variables (*n* = 102 patients); ocular variables are eye-level variables (*n* = 160 eyes). SD = standard deviation, D = diopters, UDVA = uncorrected distance visual acuity, CDVA = corrected distance visual acuity, CCT = central corneal thickness, RST = residual stromal thickness, OZ = optical zone, and IOP = intraocular pressure.

**Table 2:** Refractive Astigmatism Subgroups.

Group	Preoperative Magnitude (D)	Percentage (Eyes)
Group 1	2.00 to 2.75 D	85.0% (136/160)
Group 2	3.00 to 3.75 D	10.6% (17/160)
Group 3	4.00 to 5.00 D	4.4% (7/160)

D = diopters.

**Table 3:** Refractive Astigmatism by Preoperative Astigmatism Subgroup.

Group	Preoperative (D) (Mean ± SD)	1-Month (D) (Mean ± SD)	3 Months (D) (Mean ± SD)	Eyes with Residual Cylinder ≤0.50 D at 3 Months, *n*/*N* (%; 95% CI)
Group 1	−2.33 ± 0.28	−0.50 ± 0.26	−0.53 ± 0.27	95/136 (69.9; 61.7–76.9)
Group 2	−3.25 ± 0.27	−0.44 ± 0.33	−0.47 ± 0.34	11/17 (64.7; 41.3–82.7)
Group 3	−4.32 ± 0.35	−0.75 ± 0.46	−0.61 ± 0.38	5/7 (71.4; 35.9–91.8)

D = diopters; SD = standard deviation; CI = confidence interval. Group 1, 2.00 to 2.75 D; Group 2, 3.00 to 3.75 D; Group 3, 4.00 to 5.00 D. Percentages indicate the proportion of eyes with a residual refractive cylinder ≤0.50 D at 3 months. The 95% confidence intervals for proportions were calculated using the Wilson method.

**Table 4:** Effect of Preoperative Cylinder Magnitude in Multivariable GEE Models for 3-Month Astigmatic Outcomes.

Parameter	B	Std. Error	95% Wald Confidence Interval		Hypothesis Test		
			Lower	Upper	Wald Chi-Square	df	Sig.
DV	−0.015	0.0470	−0.107	0.077	0.103	1	0.749
Residual refractive cylinder	0.016	0.0467	−0.075	0.108	0.119	1	0.730
AE	0.946	0.4157	0.132	1.761	5.182	1	0.023

DV = difference vector; AE = angle of error; Std. = standard; df = degrees of freedom; Sig. = statistical significance. Estimates are from multivariable GEE models adjusted for age, sex, preoperative spherical equivalent, optical zone diameter, estimated residual stromal thickness, absolute intraoperative cyclotorsion, and lenticule decentration magnitude. Additional model estimates are provided in Supplementary Table S1.

## Data Availability

The raw data supporting the conclusions of this article will be made available by the authors on reasonable request, subject to institutional approval and applicable privacy regulations.

## Supplementary Materials

The following supporting information can be downloaded at: https://www.mdpi.com/article/10.3390/vision10030045/s1, Supplementary Table S1: Additional multivariable GEE estimates for 3-month astigmatic vector and residual refractive outcomes.

## Abbreviations

AE, angle of error; CDVA, corrected distance visual acuity; CI, correction index; DV, difference vector; GEE, generalized estimating equations; KLEx, keratorefractive lenticule extraction; SEQ, spherical equivalent; SIA, surgically induced astigmatism; SMILE, small-incision lenticule extraction; TIA, target-induced astigmatism; UDVA, uncorrected distance visual acuity.
