# Supplementary material for: Refractive and Vector Outcomes of SMILE Pro with the VISUMAX 800 for High Astigmatism
Source: Vision (Basel). 2026 Jul 17;10(3):45. doi: 10.3390/vision10030045 (PMC13398261; doi:10.3390/vision10030045)
Supplement: Supplementary file 1 [file vision-10-00045-s001.zip › vision-4386799-supplementary.pdf]

Supplementary Table S1. Additional multivariable GEE estimates for 3-month astigmatic vector and residual refractive outcomes

Outcome	Predictor	B	Std. Error	95% Wald Confidence Interval		Hypothesis Test		
				Lower	Upper	Wald χ^2	df	p value
Difference vector (D)	Age, per year	0.004	0.0046	-0.005	0.013	0.687	1	0.407
Difference vector (D)	Female sex versus male	-0.053	0.0359	-0.123	0.018	2.141	1	0.143
Difference vector (D)	Preoperative SEQ, per 1 D	0.004	0.0116	-0.019	0.026	0.096	1	0.757
Difference vector (D)	Optical zone, per 0.1 mm	-0.005	0.0087	-0.022	0.012	0.388	1	0.533
Difference vector (D)	Estimated RST, per 10 µm	0.004	0.0096	-0.014	0.023	0.208	1	0.648
Difference vector (D)	Absolute cyclotorsion, per 1°	0.003	0.0091	-0.015	0.021	0.109	1	0.742
Difference vector (D)	Lenticule decentration, per 0.1 mm	0.022	0.0236	-0.025	0.068	0.834	1	0.361
Absolute angle of error (°)	Age, per year	0.056	0.0549	-0.051	0.164	1.054	1	0.305

Absolute angle of error (°)	Female sex versus male	-0.603	0.4810	-1.546	0.340	1.572	1	0.210
Absolute angle of error (°)	Preoperative SEQ, per 1 D	0.180	0.1330	-0.081	0.440	1.822	1	0.177
Absolute angle of error (°)	Optical zone, per 0.1 mm	-0.080	0.1162	-0.308	0.147	0.477	1	0.490
Absolute angle of error (°)	Estimated RST, per 10 µm	-0.033	0.1159	-0.260	0.194	0.081	1	0.776
Absolute angle of error (°)	Absolute cyclotorsion, per 1°	0.190	0.1340	-0.073	0.453	2.012	1	0.156
Absolute angle of error (°)	Lenticule decentration, per 0.1 mm	0.392	0.3691	-0.332	1.115	1.127	1	0.288
Residual refractive cylinder magnitude (D)	Age, per year	0.004	0.0046	-0.005	0.013	0.726	1	0.394
Residual refractive cylinder magnitude (D)	Female sex versus male	-0.054	0.0358	-0.124	0.016	2.300	1	0.129
Residual refractive cylinder magnitude (D)	Preoperative SEQ, per 1 D	0.003	0.0115	-0.019	0.026	0.081	1	0.776

Residual refractive cylinder magnitude (D)	Optical zone, per 0.1 mm	-0.005	0.0086	-0.022	0.011	0.400	1	0.527
Residual refractive cylinder magnitude (D)	Estimated RST, per 10 µm	0.004	0.0095	-0.015	0.023	0.184	1	0.668
Residual refractive cylinder magnitude (D)	Absolute cyclotorsion, per 1°	0.003	0.0090	-0.014	0.021	0.131	1	0.718
Residual refractive cylinder magnitude (D)	Lenticule decentration, per 0.1 mm	0.021	0.0235	-0.025	0.067	0.806	1	0.369

GEE = generalized estimating equation; CI = confidence interval; D = diopters; SEQ = spherical equivalent; RST = residual stromal thickness. Models used an exchangeable working correlation structure with robust standard errors to account for inter-eye correlation. Male sex was used as the reference category. Optical zone diameter and lenticule decentration were scaled per 0.1 mm, and estimated residual stromal thickness was scaled per 10 µm. Preoperative cylinder magnitude was included in the same multivariable models and is reported in Table 4; therefore, it is not repeated here.
